# tRF‐3a‐Pro: A Transfer RNA‐Derived Small RNA as a Novel Biomarker for Diagnosis of Hepatitis B Virus‐Related Hepatocellular Carcinoma

**DOI:** 10.1111/cpr.70006

**Published:** 2025-02-24

**Authors:** Jingyi Si, Yanting Zou, Yifan Gao, Jia Chen, Wei Jiang, Xizhong Shen, Changfeng Zhu, Qunyan Yao

**Affiliations:** ^1^ Department of Gastroenterology and Hepatology, Zhongshan Hospital Fudan University Shanghai China; ^2^ Department of Gastroenterology and Hepatology, Zhongshan Hospital (Xiamen) Fudan University Xiamen China; ^3^ Shanghai Institute of Liver Diseases Shanghai China

**Keywords:** biomarker, hepatocellular carcinoma, transfer RNA‐derived small RNAs, tRF‐3a‐Pro

## Abstract

Hepatocellular carcinoma (HCC) is one of the most challenging malignancies of the digestive system. Screening for novel biomarkers and therapeutic targets is a promising strategy to enhance HCC prognosis. Recently, liquid biopsy with circulating nucleic acids as the detection targets has attracted much attention in the field of early screening of tumours. However, the diagnostic value and biological functions of transfer RNA‐derived small RNAs (tsRNAs) in serum, particularly in HCC, remains unknown. In this study, we characterised the expression profile of tsRNAs in hepatitis B virus (HBV)‐related HCC, and confirmed the diagnostic potential of serum tRF‐3a‐Pro. On this basis, we established a diagnostic model that integrates tRF‐3a‐Pro with the classic HCC biomarker alpha‐fetoprotein (AFP) through logistic regression analysis. Besides, both in vivo and in vitro experiments demonstrated that tRF‐3a‐Pro, a highly expressed tsRNA, promotes HCC cell proliferation. These findings suggested that tRF‐3a‐Pro could serve as a novel biomarker for HBV‐related HCC.

## Graphical abstract

tRF‐3a is the main tsRNA subtype up‐regulated in HBV‐related HCC. tRF‐3a‐Pro is significantly up‐regulated in HBV‐related HCC tumour than that in paratumour tissues. The level of tRF‐3a‐Pro in serum is a promising candidate biomarker for HCC diagnosis. tRF‐3a‐Pro promotes cell proliferation and regulates cell cycle of HCC.

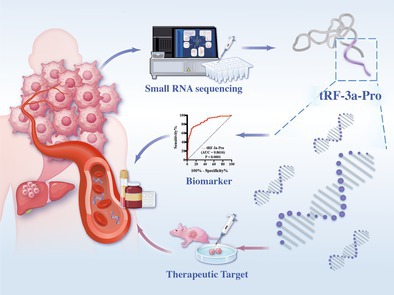

## 1 Introduction

Hepatocellular carcinoma (HCC) represents one of the most formidable challenges in digestive system malignancies, characterised by its high morbidity and mortality, with chronic infection of hepatitis B virus (HBV) being one of the main causes. This devastating disease has caused a serious burden on the economy and medical system of China [1, 2, 3]. While surgical resection remains the gold standard treatment for early‐stage HCC, the insidious progression of the disease often results in delayed diagnosis, with a majority of patients diagnosed at advanced stages [4]. Despite significant advances in therapeutic approaches, including intervention therapy, targeted therapy, and immunotherapy for patients who are not surgical candidates, the overall prognosis of HCC remains disappointingly poor [5, 6, 7]. This pressing clinical challenge underscores the urgent need to identify effective diagnostic biomarkers to improve patient outcomes through early detection and intervention.

The landscape of cancer research has been transformed by recent technological breakthroughs in sequencing methodologies and bioinformatic analyses, particularly in our understanding of non‐coding RNAs (ncRNAs) and their crucial roles in cellular processes and disease pathogenesis [8, 9, 10]. For HCC researches, extensive studies have focused on various ncRNAs, particularly microRNAs (miRNAs), long non‐coding RNAs (lncRNAs), and circular RNAs (circRNAs) [11, 12, 13]. Besides, transfer RNA‐derived small RNAs (tsRNAs) have emerged as a novel and promising area of investigation, offering unique insights into cancer biology and potential therapeutic targets.

tsRNAs originate from the precise enzymatic cleavage of mature or precursor tRNAs [14, 15]. This cleavage results in several distinct types of tsRNAs, mainly including tRNA‐derived fragments (tRFs) and tRNA‐derived stress‐induced RNAs (tiRNAs) [16, 17]. With conserved sequences and abundant modifications, tsRNAs exist in various bodily fluids and have potential value for the diagnosis and prognostic prediction of various diseases, especially malignant tumours [18, 19, 20, 21]. Furthermore, the growing understanding of tsRNAs biological functions also indicates their prospects in basic research and anti‐tumour therapies. For instance, 5'tiRNA‐His‐GTG, a tsRNA regulated by the hypoxia‐inducible factor 1 subunit alpha (HIF1α)/ANG axis, was identified to play an oncogenic role in the progression of colorectal cancer, by suppressing hippo signalling [22]. Roy et al. showed that the 3’‐tRF fragment from tRNA‐Gly‐GCC may contribute to reproductive centre‐derived lymphoma pathogenesis by regulating the DNA‐dynamics gene RPA1 [23]. While tsRNA research is extensive in solid and haematological tumours, further investigation is warranted in HCC.

In our present investigation, we conducted a comprehensive analysis of tsRNA expression profiles in HBV‐related HCCs, employing advanced sequencing technologies and rigorous bioinformatic analyses. Our findings revealed significant upregulation of tRF‐3a subtype in HCC. Notably, subsequent validation studies in patient serum samples demonstrated elevated levels of tRF‐3a‐Pro. This discovery highlights tRF‐3a‐Pro as a promising candidate biomarker for diagnosis of HBV‐related HCC, potentially offering a new avenue for early detection and monitoring of disease progression.

## 2 Materials and Methods

### 2.1 Clinical Samples

The cohort included healthy controls (HC) and patients with liver cirrhosis (LC) and HBV‐related HCC. The 33 pairs of tumour tissues and matched paratumour tissues in this study were from patients who underwent partial hepatectomy in Zhongshan Hospital Affiliated with Fudan University. The tissues used for RNA extraction were divided into cryogenic tubes, frozen with liquid nitrogen and stored in a −80°C refrigerator. The stained tissue was immersed in 4% paraformaldehyde fixative and then paraffin‐embedded sections were performed. All HCC patients were diagnosed on the basis of histopathology. Serum samples were obtained from patients with HCC (*n* = 117), patients with LC (*n* = 59) and HC (*n* = 50) who came to Zhongshan Hospital for medical treatment or physical examination from January 2022 to January 2023. SST separation gel and coagulator (Becton Dickinson, USA) were used to collect venous blood from all subjects included in the study. After centrifugation at 3000 rpm for 10 min, the supernatant was divided into tubes and stored in the refrigerator at −80°C until use. This study had been approved by the Ethics Committee of Zhongshan Hospital Affiliated to Fudan University (B2022‐482R2), and all patients signed informed consent.

### 2.2 Small RNA Sequencing

Total RNA of tissues was extracted using TRIzol (Vazyme, China) and pretreated using RNA Pretreatment (GenSeq Inc., China) to remove RNA modification. RNA library was constructed using GenSeq Small RNA Library Prep Kit (GenSeq Inc.). The target fragment of 135–160 bp (corresponding to the small RNA size of 15–40 nt) was extracted and purified by polyacrylamide gel electrophoresis (PAGE). The quantity measurement of the completed library was performed using the 2100 bioanalyser (Agilent, USA). Sequencing was performed using the Illumina HiSeq (Illumina, USA). The expression matrix was obtained from the raw reads after quality control, uncoupling and standardisation. The subsequent analysis and mapping of differentially expressed tsRNAs were completed with the help of R software.

### 2.3 Quantitative Real‐Time Polymerase Chain Reaction (qRT‐PCR)

Small RNAs in serum and culture medium were isolated using miRcute miRNA extraction and separation kit DP501 (TIANGEN, China) per the manufacturer's protocol. Total RNA/small RNA was reverse transcribed into cDNA using the ABScript II cDNA First Strand Synthesis Kit (ABclonal, China) following the manufacturer's protocol. Then the tsRNA levels were quantified with a Hieff UNICON Universal Blue qPCR SYBR Master Mix (Yeason, China) on a Q5 Real‐Time PCR System (Applied Biosystems, USA). Relative tsRNAs levels were calculated using the 2^−ΔΔCT^ method, with U6 as the endogenous gene or cel‐mir‐39 (a nematode‐derived miRNA) as exogenous reference gene. Primer sequences for reverse transcription and qRT‐PCR were provided in Tables S1 and S2.

### 2.4 Fluorescence In Situ Hybridisation

Fluorescence in situ hybridisation (FISH) was conducted on slides containing HCC tissues and matched paratumour tissues using an oligonucleotide probe against tRF‐3a‐Pro with 5′‐SA‐biotin‐label. Hybridisation process was conducted by RNA FISH kit (GenePharma, China) following the manufacturer's protocol.

### 2.5 Cell Transfection

Lentiviruses for tRF‐3a‐Pro overexpression and knockdown, along with the corresponding controls, were constructed by Genomeditech (Shanghai, China). After seeding in 6‐well plates for 24 h, the cells were treated with diluted virus solution. After another 24 h, the virus‐containing medium was replaced with DMEM complete culture medium supplemented with 2 μg/mL puromycin for drug resistance screening of the stably transformed cells. All the interfering sequences are listed in Table S3.

### 2.6 Cell Proliferation, Colony Formation, and Flow Cytometry Assay of Cell Cycle

Transfected cells were seeded in 96‐well plates with a density of 2000 cells per well for Cell Counting Kit 8 (CCK8) measurements, with a CCK8 kit (Beyotime, China) according to the manufacturer's instructions. Each assay was performed in quintuplicate. Cell proliferation was also assessed using a 5‐ethynyl‐2‐deoxyuridine (EdU) assay with an EdU kit (Beyotime, China), repeated five times. For the colony formation assay, transfected cells (1000 cells per well) were cultured in 6‐well plates for 14 days. Afterwards, they were fixed with 4% formaldehyde and stained with crystal violet (Yeason, China) following the manufacturer's instructions. Each well was photographed, and the number of colonies was counted using ImageJ software. This assay was repeated three times. For the cell cycle assay, treated cells were fixed in 75% alcohol overnight at 4°C, and a Cell Cycle Analysis Kit (Beyotime, China) was utilised for propidium iodide (PI) staining, with each assay repeated three times.

### 2.7 Immunohistochemistry (IHC) Staining

The xenografted tumours were fixed with paraformaldehyde and subsequently embedded in paraffin. Sections (4 μm thick) were then blocked with BSA and incubated successively with the primary antibody, Ki67 Rabbit mAb (ABclonal, China) and secondary antibodies, HRP Goat Anti‐Rabbit IgG (H + L) (ABclonal, China). Finally, the sections were stained with DAB reagent and haematoxylin, and then sealed for observation with the microscope. The percentage of positive tumours and the intensity of cell staining were assessed based on three randomly selected regions per section.

### 2.8 Subcutaneous Tumorigenesis Assay

Five‐week‐old BALB/c Nude mice were selected for the tumorigenesis assay. The mice were obtained from Cavens Biological Technology (Jiangsu, China) and were maintained at the Experimental Animal Center of Zhongshan Hospital, Fudan University. The mice were randomly assigned to groups (*n* = 5). A total of 5 × 10^6 MHCC97H cells stably transfected with sh‐NC or control sh‐Pro were subcutaneously injected into the right upper back of the nude mice. Tumour volumes were measured weekly. After 3 weeks, the tumours were excised for weight measurement and immunohistochemistry (IHC) staining. All animal experiments were approved by the Institutional Animal Care and Use Committee of Zhongshan Hospital, Fudan University.

### 2.9 Statistical Analysis

SPSS 24.0, Prism 9.0, and R 4.2.0 were used for statistical analysis. The expression matrix of sequencing data was used to determine the source of tsRNA according to the tRNA sequences in the GtRNAdb database, and to match the tsRNA subclasses. The expression matrix after ID transformation was analysed by “edgeR”, “FactoMineR” package, and “ggplot2” package. For qRT‐PCR data and clinical data of subjects in three groups, the Student's t statistical method was used to test continuous variables, Chi‐square test was used to test categorical variables and the Pearson correlation coefficient was used to test the correlation between the two groups. Logistic regression was used to establish a diagnostic model of alpha‐fetoprotein (AFP) and tRF‐3a‐Pro. The receiver operating characteristic (ROC) curve was drawn and the area under the curve (AUC) was used to evaluate the diagnostic efficiency. Youden index (sensitivity + specificity—1) was used to calculate the cut‐off value. The corresponding sensitivity and specificity were recorded with the maximum Youden index. DeLong's test was used to compare the difference of ROC curves between different diagnostic methods. A *p* value of less than 0.05 was considered statistically significant.

## 3 Results

### 3.1 Differential Expression of tsRNAs in HBV‐Related HCC Tumour and Paratumour Tissues

**FIGURE 1:**
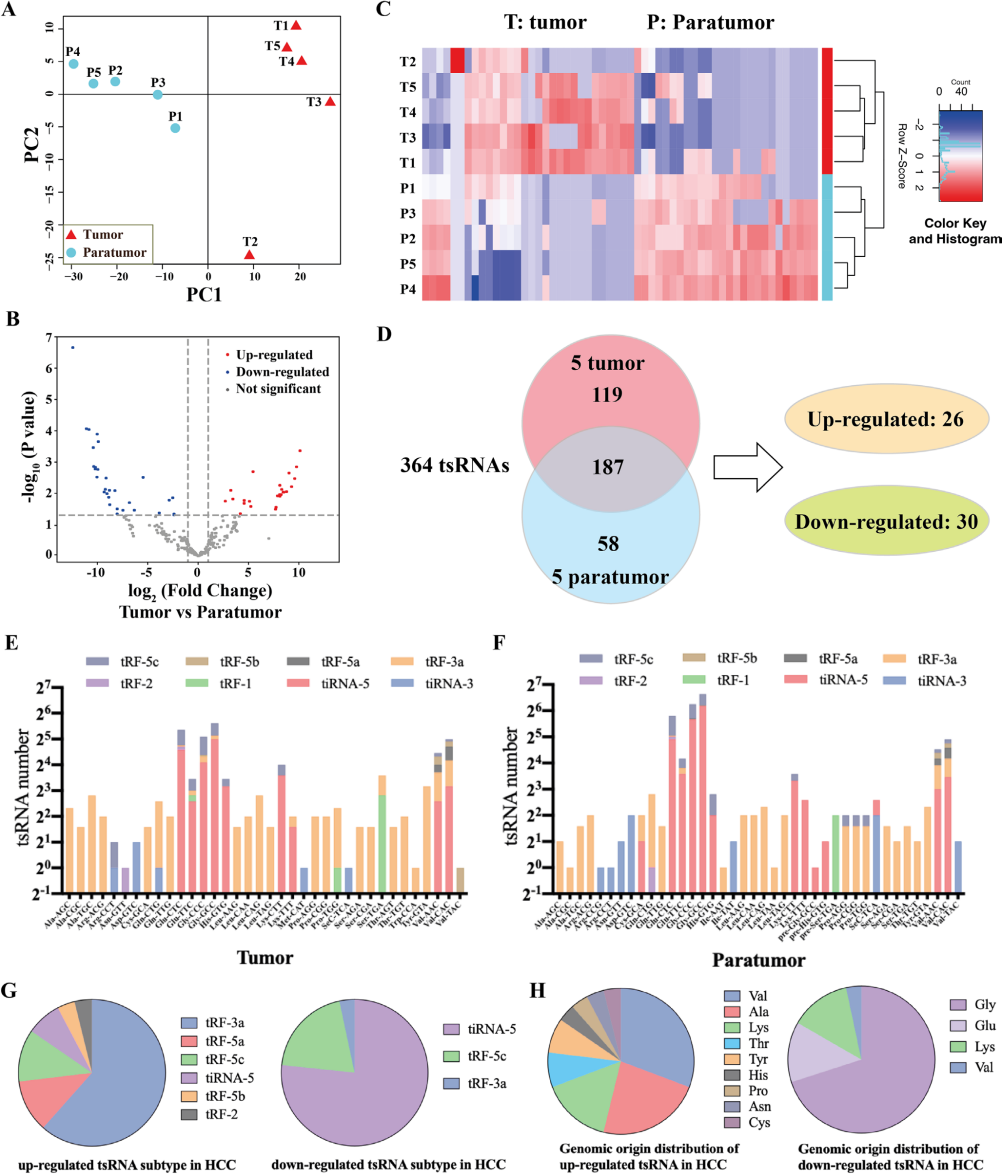
The expression profiles of tsRNAs in HCC tumours and paratumour tissues. (A) PCA of tsRNA expression in HCC and paratumour tissues. (B) Volcano plot of differential expression of tsRNA between 5 pairs of HCC tumour and paratumour tissues. (C) Hierarchical clustering heatmap of differentially expressed tsRNAs in HCC tissues compared to paratumour tissues. (D) Venn diagram showing the overlapping of tsRNAs in tumour and paratumour tissues of HCC. (E) The distribution of tsRNA subtypes in tumour and (F) paratumour tissues of HCC. (G) The distribution of up‐regulated and down‐regulated tsRNA subtypes in HCC tissues. (H) The distribution of tRNA sources for up‐regulated and down‐regulated tsRNA in HCC tissues.

To investigate the expression profiles in HBV‐related HCC tumour and paratumour tissues, RNA extraction and tsRNA sequencing were performed in 5 pairs of tissues. Principal component analysis (PCA) showed that dots presenting tumour (red) and paratumour (blue) tissues were clearly grouped, indicating that there were significant differences in tsRNA expression in HCC tumour and paratumour tissues (Figure 1A). As a result, a total of 394 tsRNAs were obtained (Figure 1B). Significance was set at uncorrected *p* < 0.05 for broad pattern identification. A log_2_ |fold change| threshold was set at > 1. A total of 26 tsRNAs were up‐regulated and 30 tsRNAs were down‐regulated in HCC tissues compared with paratumour tissues (Figure 1C).

In order to fully characterise the tsRNA expression profiles, the subclasses and origin of tsRNAs were analysed. The highest proportion of tsRNA subtype obtained from sequencing results was tRF‐3a, followed by tiRNA‐5 (Figure 1E,F). Among the tsRNAs with significant differences in expression, tRF‐3a was the most up‐regulated tsRNA subtype in HCC tissues, and tiRNA‐5 was the most down‐regulated tsRNA subtype, indicating that tRF‐3a may be a potential tumour‐promoting tsRNA subtype (Figure 1G). Additionally, up‐regulated tsRNAs were mainly derived from valine tRNAs, and down‐regulated tsRNAs were mainly derived from glycine tRNAs (Figure 1H).

### 3.2 Proline‐tRNA‐Derived tRF‐3a Was Up‐Regulated in Tumour Tissues and Serum of HBV‐Related HCC Patients

**FIGURE 2:**
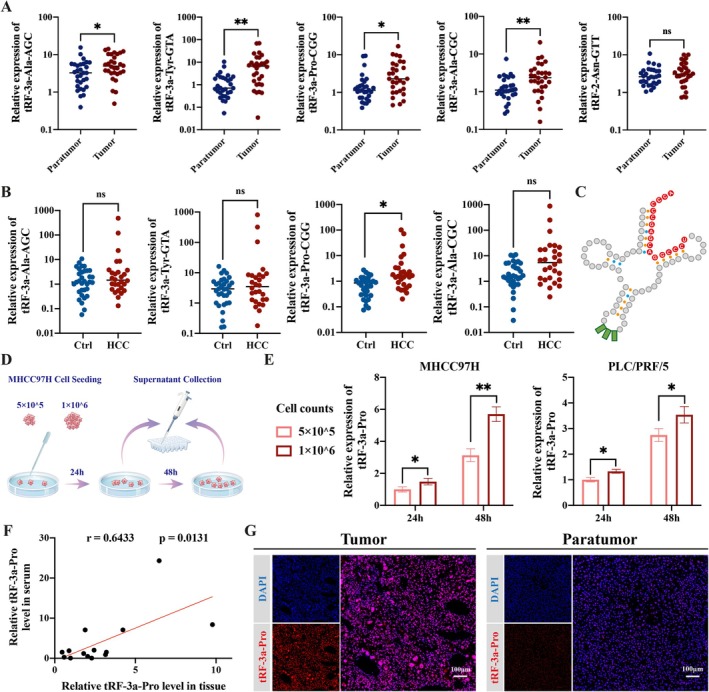
Levels of proline‐tRNA‐derived tRF‐3a in tumour tissues and serum of HBV‐related HCC patients. (A) qRT‐PCR validation of differential expression of candidate tsRNAs in HCC tumour and paratumour tissues (*n* = 28). (B) qRT‐PCR validation of differential expression of candidate tsRNAs in serum of HCC patients (*n* = 28) and control groups (*n* = 33). (C) Biological source prediction of tRF‐3a‐Pro. (D) Schematic diagram of tRF‐3a‐Pro detection in cell supernatant. (E) The concentration of tRF‐3a‐Pro was secreted into medium with different MHCC97H cell numbers and culture times. (F) Pearson correlation coefficient analysis of tRF‐3a‐Pro content in paired HCC tissues and serum (*n* = 14). (G) Localisation of tRF‐3a‐Pro in HCC tumour and paratumour tissues. Scale bar = 100 μm. ns, no significant, *p < 0.05, **p < 0.01.

In order to further verify the differential expression of tsRNA in HCC tumour and paratumour tissues, we selected the top 5 up‐regulated tsRNAs, and performed RNA extraction and qRT‐PCR analysis in 28 pairs of HCC tumour and paratumour tissues. It showed that the top four up‐regulated tsRNAs in the sequencing results, tRF‐3a‐Ala‐AGC, tRF‐3a‐Tyr‐GTA, tRF‐3a‐Pro‐CGG and tRF‐3a‐Ala‐CGC, were significantly up‐regulated in HCC tumour tissues. However, the expression of tRF‐2‐Asn‐GTT was not significantly up‐regulated (Figure 2A). To make sure whether the above tsRNAs also have abnormal expression in the serum of HBV‐related HCC patients, we first conducted a small‐scale analysis. Compared with the control group (16 LC patients and 17 healthy individuals), tRF‐3a‐Pro‐CGG was significantly up‐regulated in the serum of 28 HCC patients. However, the contents of the other three tsRNAs (tRF‐3a‐Ala‐AGC, tRF‐3a‐Tyr‐GTA, and tRF‐3a‐Ala‐CGC) were not significantly distinguished (Figure 2B).

Since each tsRNA sequence only matched the first tRNA in the GtRNAdb website during the ID transformation of the sequencing data expression matrix, it cannot be determined whether a tsRNA is generated by the specific tRNA. Proline tsRNAs include tRNA‐Pro‐AGG and tRNA‐Pro‐UGG in addition to tRNA‐Pro‐CGG, which are distinguished by different anti‐codons. The sequence of tRF‐3a was found to be conserved among proline tsRNAs after alignment (Figure 2C). The result suggested that tRF‐3A‐Pro‐CGG (hereafter referred to as tRF‐3a‐Pro) may be derived from the 3 types of proline tRNAs above. To further explore whether the increased tRF‐3a‐Pro was released from HCC cells, tRF‐3a‐Pro expression was investigated in MHCC97H and PLC/PRF/5 culture medium over 24 and 48 h (Figure 2D). We found that tRF‐3a‐Pro expression was directly proportional to cell number and culture time (Figure 2E). In addition, tRF‐3a‐Pro level in serum was significantly positively correlated with that in paired HCC tissues from the same patient (*r* = 0.6433) (Figure 2F). The above results indicated that tRF‐3a‐Pro in serum may be derived from tumour tissues. To further investigate the tissue distribution of tRF‐3a‐Pro, we performed FISH on paraffin sections. Figure 2G showed that tRF‐3a‐Pro is mainly distributed in the nucleus of HCC tumour cells.

### 3.3 Diagnostic Value of tRF‐3a‐Pro for HBV‐Related HCC

Next, the expression of the tRF‐3a‐Pro was further analysed in a larger cohort of serum samples (117 HCC patients, 59 LC patients, and 50 healthy individuals), whose information was shown in Table 1. Alanine aminotransferase (ALT) levels were similar among HCC patients and control groups. However, individuals in control groups were younger and more likely to be women.

**TABLE 1:** Patient characteristics.

	HCC (*n* = 117)	Liver cirrhosis(*n* = 59)	Healthy controls (*n* = 50)	*p*
	*n* (%)	*n* (%)	*n* (%)	
Age, years				< 0.0001
≧ 60	60 (51.28)	25 (42.37)	5 (10.00)	
< 60	57 (48.72)	34 (57.63)	45 (90.00)	
Gender				< 0.0001
Male	103 (88.03)	40 (67.80)	23 (46.00)	
Female	14 (11.97)	19 (32.20)	27 (54.00)	
ALT				0.8896
≦ 40 U/L	97 (82.91)	49 (83.05)	45 (90.00)	
> 40 U/L	20 (17.09)	10 (16.95)	5 (10.00)	
AFP				< 0.0001
< 20 μg/L	63 (53.85)	58 (98.32)	50 (100.00)	
≧ 20 μg/L	54 (46.15)	1 (1.70)	0	
Diameter of tumour				
< 3 cm	41 (35.04)	—	—	
3–5 cm	46 (39.32)	—	—	
> 5 cm	30 (25.64)	—	—	
Number of tumours				
= 1	95 (81.20)	—	—	
> 1	22 (18.80)	—	—	
BCLC stage				
0	26 (22.22)	—	—	
A	71 (60.68)	—	—	
B	9 (7.69)	—	—	
C	11 (9.41)	—	—	
CNLC stage				
Ia	69 (58.97)	—	—	
Ib	32 (27.35)	—	—	
IIa	6 (5.13)	—	—	
IIb	6 (5.13)	—	—	
IIIa	4 (3.42)	—	—	

Abbreviations: AFP, alpha‐fetoprotein; ALT, alanine aminotransferase; BCLC, barcelona clinic liver cancer; CNLC, China liver cancer staging;HCC, hepatocellular carcinoma.

**FIGURE 3:**
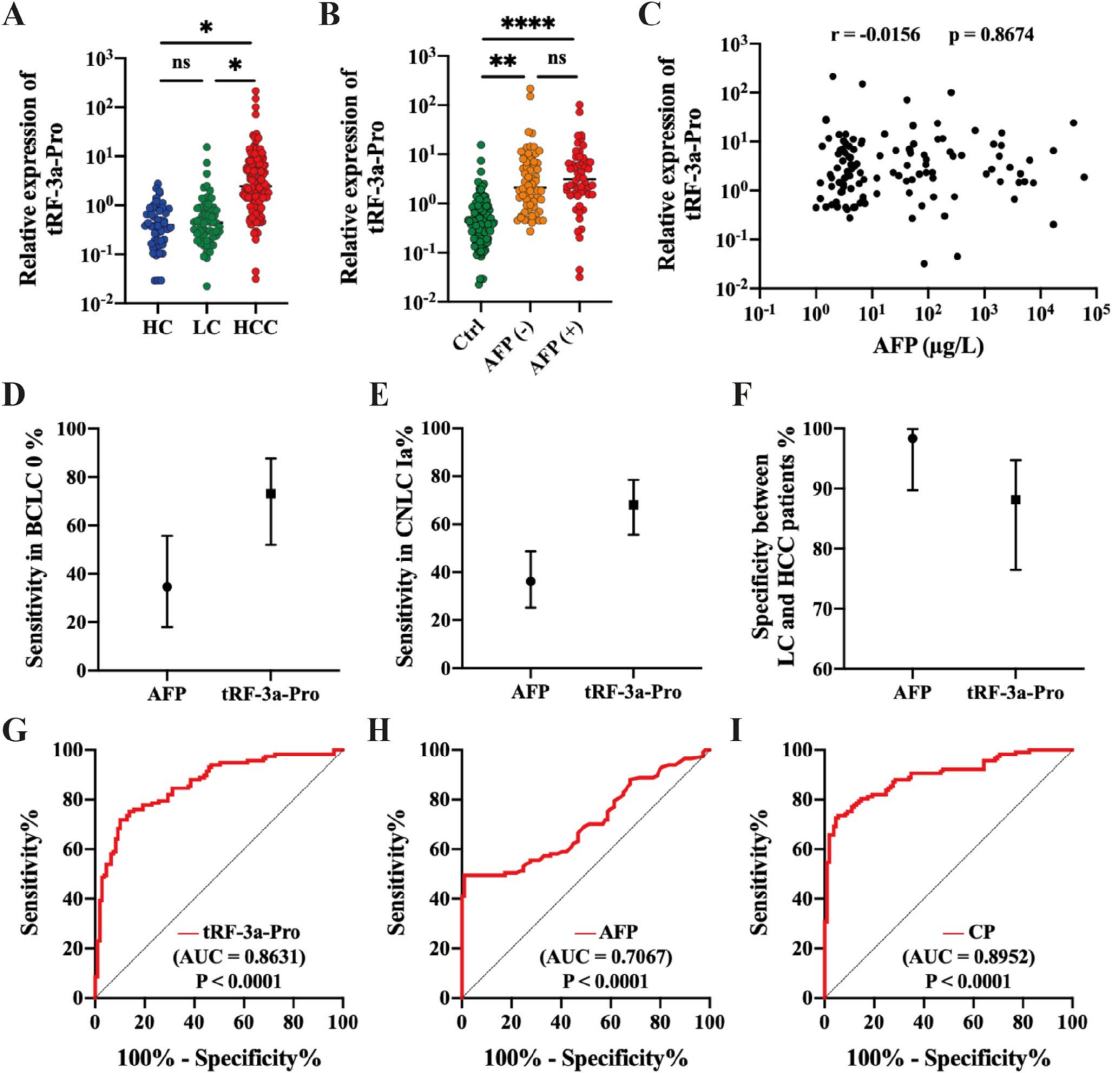
Diagnostic value of serum tRF‐3a‐Pro for HBV‐related HCC patients. (A) Serum tRF‐3a‐Pro levels in HC group (*n* = 117), LC group (*n* = 59), and HCC group (*n* = 50). (B) Serum tRF‐3a‐Pro levels in control group (*n* = 109), AFP (−) group (*n* = 63), and AFP (+) group (*n* = 54). (C) Pearson correlation between tRF‐3a‐Pro and AFP. (D) Sensitivity of AFP, and tRF‐3a‐Pro in distinguishing BCLC stage 0 HCC. (E) Sensitivity of AFP, and tRF‐3a‐Pro in distinguishing CNLC stage Ia HCC. (F) The specificity of AFP, and tRF‐3a‐Pro in distinguishing HBV‐related HCC and LC patients. (G–I) ROC curves of tRF‐3a‐Pro, AFP, and CP for distinguishing HCC from the control group. ns, no significant, *p < 0.05, **p < 0.01, ****p < 0.0001.

The serum levels of tRF‐3a‐Pro of HCC patients were significantly higher than those of LC patients and healthy individuals respectively (Figure 3A). Although the mean serum tRF‐3a‐Pro level was higher in the cirrhosis patients than in healthy individuals, the difference between the two groups was not statistically significant (1.008 vs. 0.6014, *p* = 0.2125). Taking AFP < 20 μg/L as the threshold, the serum tRF‐3a‐Pro level in AFP‐negative HCC patients was also significantly higher than that in control groups (Figure 3B). The correlation analysis showed that there was no significant correlation between levels of serum tRF‐3a‐Pro and AFP (*p* = 0.8656) (Figure 3C).

Further analysis revealed that tRF‐3a‐Pro had higher diagnostic sensitivity for early HCC compared to AFP. For BCLC stage 0 HCC patients, the sensitivity (95% CI) of AFP was 34.62% (17.94%, 55.64%), and the sensitivity (95% CI) of tRF‐3a‐Pro was 73.08% (51.95%, 87.65%) (Figure 3D). For HCC patients at CNLC stage Ia, the sensitivity (95% CI) of AFP was 36.23% (25.26%, 48.75%), and the sensitivity (95% CI) of tRF‐3a‐Pro was 68.12% (55.67%, 78.53%) (Figure 3E). The incidence of HCC in patients with cirrhosis is much higher than that in non‐cirrhotic patients [24], however, distinguishing between patients with LC and early‐stage HCC has always been a challenge in the development of HCC biomarkers. The analysis results showed that the specificity (95% CI) of tRF‐3a‐Pro was 88.14% (76.63%, 94.70%), slightly lower than that of AFP 98.31% (89.70%, 99.91%) (Figure 3F). Therefore, the combined application of tRF‐3a‐Pro and AFP may improve the diagnostic efficiency for HCC.

ROC analysis was performed to determine the diagnostic value of tRF‐3a‐Pro. It individually showed a high diagnostic value with an AUC (95% CI) of 0.8631 (0.8153, 0.9108), exhibiting a sensitivity of 71.80% and specificity of 89.91% under a diagnostic threshold of 1.4197 (Figure 3G). Subsequently, we analysed the diagnostic efficiency of AFP, and the results showed that the AUC (95% CI) of AFP was 0.7067 (0.6388, 0.7746) (Figure 3H). In the combined analysis, the signature with tRF‐3a‐Pro and AFP has a higher AUC (95% CI), 0.8952 (0.8537, 0.9366), with the cut‐off value of −0.1011 under a logistic regression model, and combining predictors (CP) = −1.7555 + 0.5486 × tRF‐3a‐Pro + 0.0617 × AFP (Figure 3I). The diagnostic parameters including sensitivity, specificity, Youden index, positive predictive value (PPV), negative predictive value (NPV) and AUC were shown in Table 2.

**TABLE 2:** Diagnostic parameters of tRF‐3a‐Pro, AFP and CP for HCC.

	Sensitivity	Specificity	Youden	PPV	NPV	AUC (95% CI)
tRF‐3a‐Pro	0.7180	0.8991	0.6170	0.8842	0.74801	0.8631 (0.8153, 0.9108)
AFP	0.4957	0.9908	0.4866	0.9831	0.6467	0.7067 (0.6388, 0.7746)
CP	0.7265	0.9541	0.6806	0.9444	0.7647	0.8952 (0.8537, 0.9366)

Abbreviations: AFP, alpha‐fetoprotein; AUC, area under curve; CP, combining predictors; NPV, negative predictive value; PPV, positive predictive value.

**FIGURE 4:**
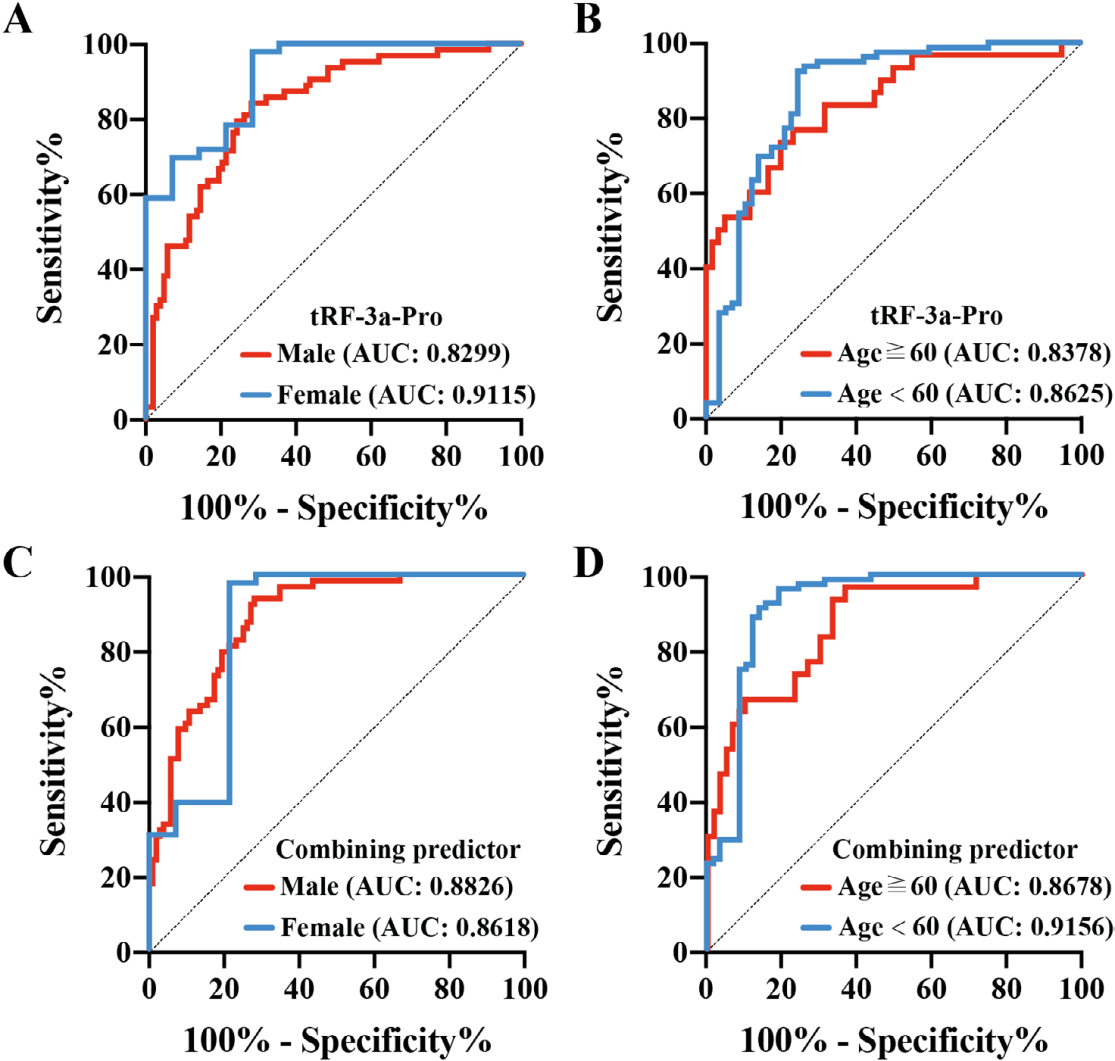
ROC curves stratified by baseline data for tRF‐3a‐Pro and CP. (A) ROC curve of tRF‐3a‐Pro stratified by gender data. (B) ROC curve of tRF‐3a‐Pro stratified by age data. (C) ROC curve of CP stratified by gender data. (D) ROC curve of CP stratified by age data.

Because of differences in baseline variables, the following analysis was plotted against control groups distributions for age and sex. The results indicated that the AUC of tRF‐3a‐Pro was 0.8299 for men and 0.9155 for women (Figure 4A), while it was 0.8378 for individuals aged 60 years or older and 0.8625 for those younger than 60 years (Figure 4B). Similarly, the AUC of CP was 0.8826 for men and 0.8618 for women (Figure 4C), and it was 0.8678 for those aged 60 years or older and 0.9156 for those under 60 (Figure 4D). There were no significant differences in the AUC of tRF‐3a‐Pro and CP between different genders, with *p*‐values of 0.1336 and 0.6726, respectively. Furthermore, no significant differences were found in the AUC of tRF‐3a‐Pro and CP across different age groups, with *p*‐values of 0.7915 and 0.3237, respectively (Table 3). These findings suggest that the variations in tRF‐3a‐Pro levels and CP values among the groups are primarily associated with the disease status of the subjects, rather than their gender and age differences.

**TABLE 3:** AUC of tRF‐3a‐Pro and CP stratified by gender and age.

		Yes AUC (95% CI)	No AUC (95% CI)	*p*
tRF‐3a‐Pro	Male sex	0.8299 (0.7666, 0.8932)	0.9115 (0.8277, 0.9953)	0.1336
	Age ≧ 60	0.8378 (0.7468, 0.9287)	0.8625 (0.7939, 0.9311)	0.6726
CP	Male sex	0.8826 (0.8329, 0.9322)	0.8618 (0.7290, 1.0000)	0.7915
	Age ≧ 60	0.8678 (0.7920, 0.9436)	0.9156 (0.8594, 0.9718)	0.3237

Abbreviations: AFP, alpha‐fetoprotein; CP, combining predictors.

### 3.4 tRF‐3a‐Pro Promotes the Proliferation of HCC Cells In Vitro

**FIGURE 5:**
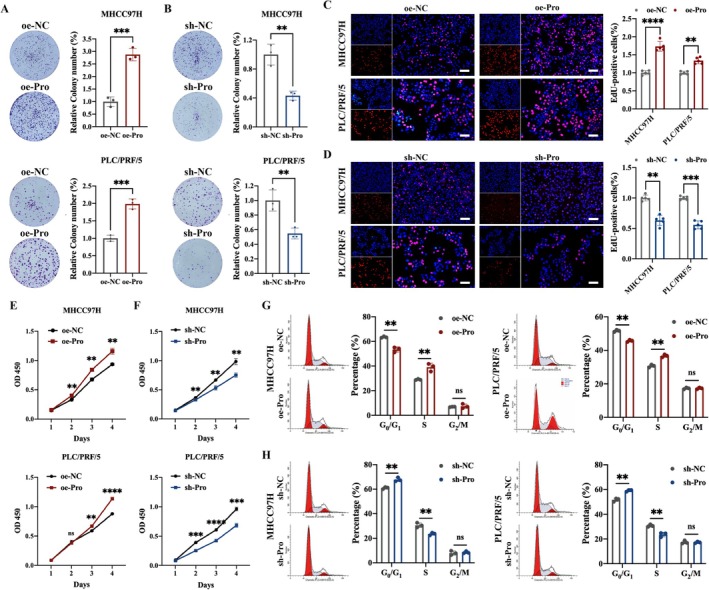
tRF‐3a‐Pro promotes the proliferation of HCC cells in vitro. (A, B) Colony formation assays were performed to evaluate cell proliferation ability of tRF‐3a‐Pro overexpression or knockdown cells. (C, D) EdU assays were performed to assess the cell proliferation ability. (E, F) CCK8 assays were applied to determine the growth curves. (G, H) Cell cycle distributions were detected by flow cytometry in tRF‐3a‐Pro overexpression or knockdown cells. All data are presented as the means ± SD of 3 or 5 independent experiments. ns, no significant, **p < 0.01, ***p < 0.001, ****p < 0.0001.

To further explore the biological effects of tRF‐3a‐Pro in HCC cells, the tRF‐3a‐Pro overexpression and knockdown models were constructed in MHCC97H and PLC/PRF/5 cells. The knockdown and overexpression efficiencies of the two cells were verified by qRT‐PCR assay (Figure S1). Colony formation assay results indicated that tRF‐3a‐Pro overexpression significantly promoted colony formation in MHCC97H and PLC/PRF/5 cells, while tRF‐3a‐Pro knockdown significantly inhibited colony formation in MHCC97H and PLC/PRF/5 cells (Figure 5A,B). Moreover, EdU assays revealed that the tRF‐3a‐Pro overexpression accelerated the proportion of MHCC97H and PLC/PRF/5 cells in the DNA synthesis phase, whereas tRF‐3a‐Pro knockdown diminished them (Figure 5C,D). CCK‐8 assay results indicated that tRF‐3a‐Pro overexpression significantly promoted the proliferation of MHCC97H and PLC/PRF/5 cells. In contrast, tRF‐3a‐Pro knockdown significantly inhibited the proliferation of MHCC97H and PLC/PRF/5 cells (Figure 5E,F). Flow cytometric assay of cell cycle distribution demonstrated that the overexpression of tRF‐3a‐Pro facilitated the G1 to S transition. However, the knockdown of tRF‐3a‐Pro dramatically increased the number of cells in the G0/G1 phase, along with a remarkable decrease in S phase cells (Figure 5G,H). To summarise, tRF‐3a‐Pro promoted proliferation and regulated the cell cycle in HCC cells in vitro.

### 3.5 Knockdown of tRF‐3a‐Pro Inhibits the Growth of HCC In Vivo

**FIGURE 6:**
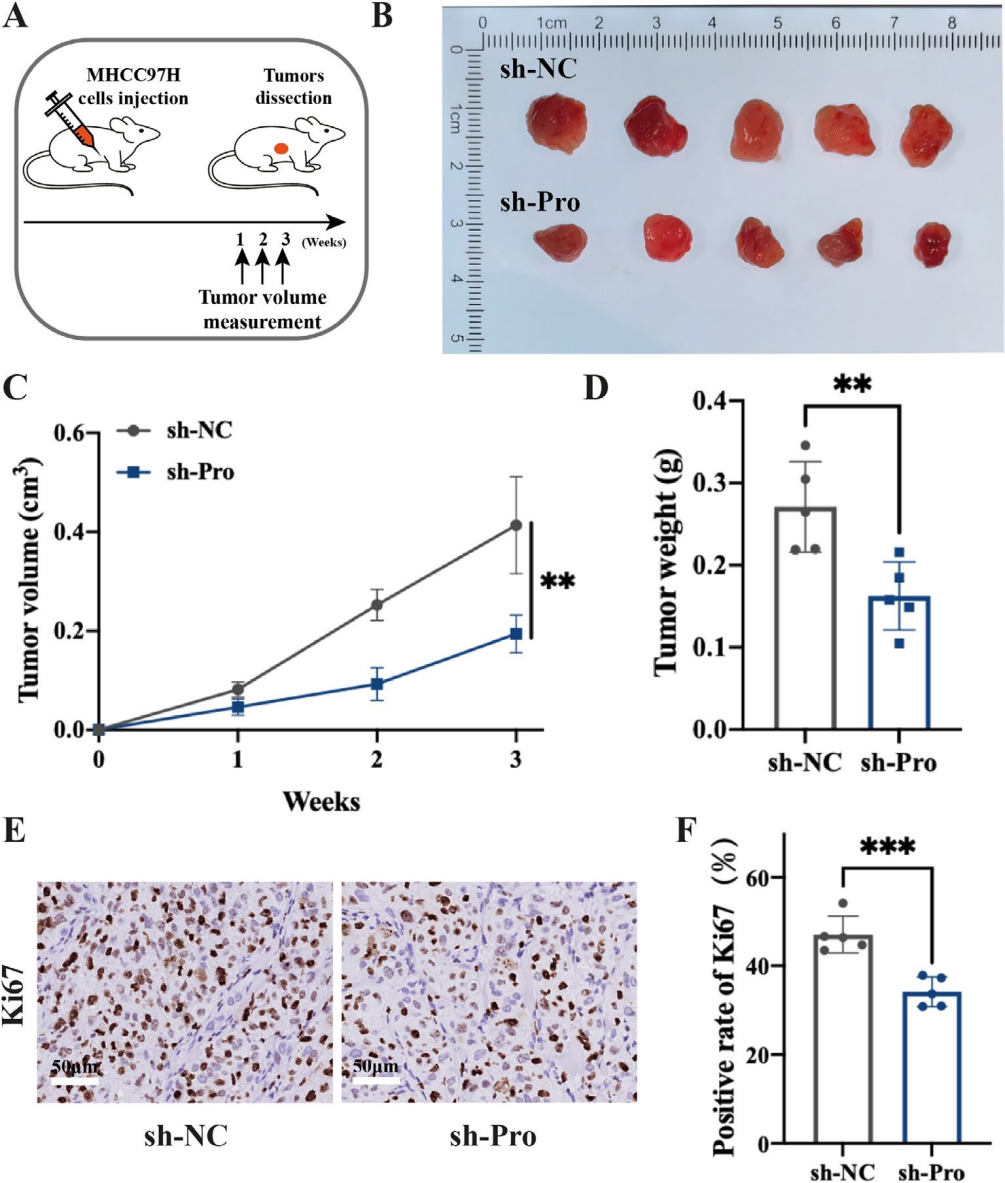
Knockdown of tRF‐3a‐Pro inhibits the growth of HCC in vivo. (A) The workflow of subcutaneous tumorigenesis. (B) Tumour image at the day 21 (*n* = 5). (C, D) Quantitative analysis of tumour weights and tumour volumes. (E) Ki67 IHC staining of subcutaneous tumour tissues. (F) Statistical analysis of Ki67 positivity rate in subcutaneous tumour tissues. The scale bar, 50 μm. Data were shown as mean ± SD. **p < 0.01, ***p < 0.001.

Next, we subcutaneously implanted the infected cells into six‐week‐old nude mice. Tumour growth was evaluated 3 weeks after cell implantation (Figure 6A). The weights or volumes of xenografted tumours were lower or smaller in the tsRNA‐knockdown group than in the control group (Figure 6B–D). In addition, immunohistochemical analysis was performed on the subcutaneous tumour tissues, and the Ki67 positivity rate in the field of view was used as an indicator to evaluate the proliferation ability of HCC cells (Figure 6E). Compared with the control group, knocking down tRF‐3a‐Pro inhibited the in vivo proliferation ability of MHCC97H cell line (Figure 6F). The subcutaneous tumorigenesis assay not only confirmed the effect of tRF‐3a‐Pro on the proliferation ability of HCC cells in vivo, but also indirectly provided theoretical support for targeted tRF‐3a‐Pro therapy for HCC.

## 4 Discussion

Selecting the correct biomarkers is essential to enhancing treatment efficacy in HCC. Therefore, there is an urgent need to develop robust non‐invasive strategies for diagnosing this globally burdensome cancer. In this study, we mainly focused on HBV‐related HCC, screened and validated the differentially expressed tsRNAs in tumour and paratumour tissues. Our findings confirmed that serum tRF‐3a‐Pro levels might serve as a novel biomarker for HBV‐related HCC. Furthermore, the combined usage of tRF‐3a‐Pro with the classic biomarker AFP could improve diagnostic value for HBV‐related HCC. This study advanced the screening and monitoring of high‐risk populations for HCC, facilitating early detection and treatment.

ncRNAs play a crucial role in regulating various fundamental biological processes and are abnormally involved in numerous human diseases, particularly malignant tumours like HCC [11, 25, 26]. tsRNAs feature conserved sequences and extensive modifications, making them detectable in various body fluids [27, 28, 29]. This gives it potential in diagnosing and prognosing malignant tumours. Our study primarily examined tsRNA expression in HBV‐related HCC tumour tissues and patients' serum. Through tsRNA sequencing, we discovered that the expression patterns of tsRNA in HCC tumour tissues significantly differed from paratumour tissues. In tumour tissues, there was predominantly upregulation of the tsRNA subtype tRF‐3a and downregulation of tiRNA‐5. The production of tsRNA is closely associated with the expression of various nucleases and tRNA modifications [15, 30, 31]. For instance, in pancreatic cancer, the upregulation of tRF‐Pro‐AGG‐004 and tRF‐Leu‐CAG‐002 might result from increased angiogenin (ANG), a nucleic acid endonuclease [28]. The elevated expression of 5’‐tRF‐GlyGCC was dependent on the upregulation of AlkB homolog3 (ALKBH3), a tRNA demethylase that promotes cleaving of tRNA to generate tsRNA [32]. Therefore, changes in tsRNA subtypes in HCC tissues may be related to these factors.

Furthermore, by assessment of serum levels of the top five elevated tsRNAs in HCC tumour tissue, we found that tRF‐3a‐Pro was also elevated in the serum of HBV‐related HCC patients. The AUC for tRF‐3a‐Pro in the HCC was 0.8631 (0.8153, 0.9108), which was significantly higher than that of the classical HCC marker AFP. The combination of AFP to tRF‐3a‐Pro increased the AUC to 0.8952 (0.8537, 0.9366). Previous studies have demonstrated that circulating tsRNAs served as powerful non‐invasive diagnostic biomarkers for various benign and malignant conditions. For instance, tRF‐Ala‐AGC‐2‐M4 was proved to have a higher expression in patients with lupus nephritis compared with healthy individuals, with an AUC of 0.758 [33]. The AUC of tRF‐19‐DRMD5112 for renal cell carcinoma was determined to be 0.8549 [34]. However, to date, no single independent tsRNA has been identified as an ideal serum biomarker for diagnosing HBV‐related HCC. Our data showed for the first time that, compared with the control group, the serum level of tRF‐3a‐Pro in the HCC group was significantly increased and that it had a strong capability to differentiate early HCC as well as distinguish between LC and HBV‐related HCC patients.

Further experiments and analyses revealed that tRF‐3a‐Pro, a proline tRNA‐derived tRNA fragment primarily localised in the nucleus, played a regulatory role in the biological functions of HCC cells. Specifically, overexpression of tRF‐3a‐Pro‐enhanceed HCC cell proliferation and facilitated their entry into the S phase of the cell division, while knockdown of tRF‐3a‐Pro inhibited these biological functions. Additionally, subcutaneous tumorigenesis experiments demonstrated that knockdown of tRF‐3a‐Pro suppressed the in vivo growth of HCC cells. These findings suggested that tRF‐3a‐Pro was not only a promising biomarker for the diagnosis of HCC but also a potential therapeutic target.

This study preliminarily confirmed the diagnostic value of tRF‐3a‐Pro for HCC and its influence on HCC biological functions. Previous research indicated that tsRNAs exerted their biological functions primarily through two mechanisms. First, due to its structural similarity to microRNAs, tsRNAs might bind to mRNA in a sequence‐dependent manner, promoting argonaute‐dependent mRNA degradation. For instance, in colorectal cancer, the downregulation of tRF‐3008A, which is derived from tRNA‐Val, disrupted the stability of FOXK1, a positive regulator of the Wnt/β‐catenin pathway, in an AGO‐dependent manner, thereby inhibiting metastasis and progression [35]. In addition, RNA‐binding proteins play a crucial role in mediating the biological functions of tsRNAs. For example, the 3′ fragment of valine tRNA was proved to directly bind to the chaperone molecule EEF1A1, facilitating its transport into the nucleus and promoting its interaction with MDM2. This interaction inhibited the downstream p53 signalling pathway and promoted gastrointestinal cancer progression [36]. Our study laid the groundwork for further in‐depth research on tRF‐3a‐Pro in HCC. Future investigations should focus on elucidating the mechanisms underlying the upregulation of tRF‐3a‐Pro in HCC and its regulatory effects on HCC cell biology, aiming to provide a more robust theoretical basis for molecular diagnosis and targeted therapy for HCC.

## Author Contributions

**Jingyi Si:** conceptualisation, data curation, investigation, methodology, validation, writing – original draft. **Yanting Zou:** data curation, resources, investigation, writing – original draft. **Yifan Gao:** investigation, methodology, resources. **Jia Chen:** investigation, methodology. **Wei Jiang:** supervision, writing – review and editing. **Xizhong Shen:** investigation, resources, supervision, writing – review and editing. **Changfeng Zhu:** project administration, resources, supervision, writing – review and editing. **Qunyan Yao:** conceptualisation, funding acquisition, project administration, supervision, writing – review and editing.

## Conflicts of Interest

The authors declare no conflicts of interest.

## Supporting information


**Data S1.** Figures.

**Data S2.** Tables.

## Data Availability

The data that support the findings of this study are available from the corresponding author upon reasonable request.
