# Supplementary material for: tRF‐3a‐Pro: A Transfer RNA‐Derived Small RNA as a Novel Biomarker for Diagnosis of Hepatitis B Virus‐Related Hepatocellular Carcinoma
Source: Cell Prolif. 2025 Feb 24;58(7):e70006. doi: 10.1111/cpr.70006 (PMC12240632; doi:10.1111/cpr.70006)
Supplement: Supplementary file 1 — Data S1. Figures. [file CPR-58-e70006-s001.docx]

Supplementary Figure


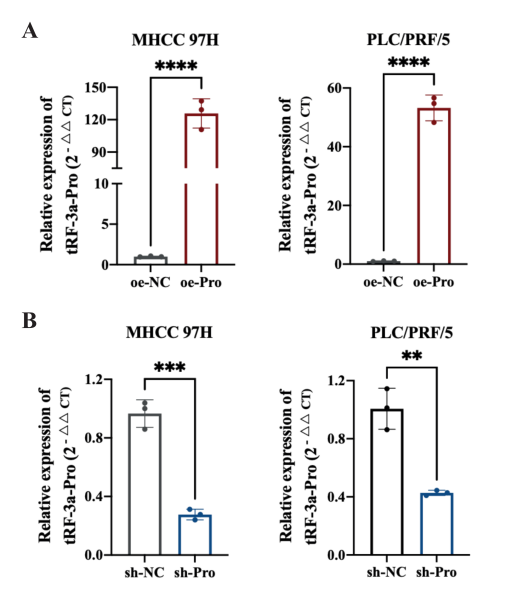


Supplementary Figure 1 The overexpression and knockdown efficiencies of tRF-3a-Pro in MHCC97H and PLC/PRF/5.

(A) qRT-PCR validation results of tRF-3a-Pro over-expression cell lines. (B) qRT-PCR validation results of tRF-3a-Pro knockdown cell lines.
