# Supplementary material for: tRF‐3a‐Pro: A Transfer RNA‐Derived Small RNA as a Novel Biomarker for Diagnosis of Hepatitis B Virus‐Related Hepatocellular Carcinoma
Source: Cell Prolif. 2025 Feb 24;58(7):e70006. doi: 10.1111/cpr.70006 (PMC12240632; doi:10.1111/cpr.70006)
Supplement: Supplementary file 2 — Data S2. Tables. [file CPR-58-e70006-s002.docx]

Supplementary Table

Supplementary Table 1 Reverse transcription primer sequences

| Name | Primer Sequence (5' - 3') |
| --- | --- |
| tRF-3a-Ala-AGC | GTCGTATCCAGTGCGTGTCGTGGAGTCGGCAATTGCACTGGATACGACTGGTGG |
| tRF-3a-Tyr-GTA | GTCGTATCCAGTGCGTGTCGTGGAGTCGGCAATTGCACTGGATACGACTGGTCC |
| tRF-3a-Pro-CGG  （tRF-3a-Pro） | GTCGTATCCAGTGCGTGTCGTGGAGTCGGCAATTGCACTGGATACGACTGGGGG |
| tRF-3a-Ala-CGC | GTCGTATCCAGTGCGTGTCGTGGAGTCGGCAATTGCACTGGATACGACTGGTGG |
| tRF-2-Asn-GTT | GTCGTATCCAGTGCGTGTCGTGGAGTCGGCAATTGCACTGGATACGACCGGTTA |
| U6 | AACGCTTCACGAATTTGCGT |
| cel-mir-39 | GTCGTATCCAGTGCGTGTCGTGGAGTCGGCAATTGCACTGGATACGACCAAGCTGA |

Supplementary Table 2 qRT-PCR primer sequences

| Name |  | Primer Sequence (5' - 3') |
| --- | --- | --- |
| tRF-3a-Ala-AGC | Forward | GGGCCCCGGCACCT |
|  | Reverse | CAGTGCGTGTCGTGGAGT |
| tRF-3a-Tyr-GTA | Forward | GGGTCCGGCTCGGA |
|  | Reverse | CAGTGCGTGTCGTGGAGT |
| tRF-3a-Pro-CGG  （tRF-3a-Pro） | Forward | GGGTCCCGGACGAG |
|  | Reverse | CAGTGCGTGTCGTGGAGT |
| tRF-3a-Ala-CGC | Forward | GGGCCCGGCATCT |
|  | Reverse | CAGTGCGTGTCGTGGAGT |
| tRF-2-Asn-GTT | Forward | GGGTTCGGCTGT |
|  | Reverse | CAGTGCGTGTCGTGGAGT |
| U6 | Forward | CTCGCTTCGGCAGCACA |
|  | Reverse | AACGCTTCACGAATTTGCGT |
| cel-mir-39 | Forward | GGGTCACCGGGTGTAAA |
|  | Reverse | CAGTGCGTGTCGTGGAGT |

Supplementary Table 3 Fluorescence in situ hybridization probe sequence

| Name | Oligonucleotide sequences (5' - 3') |
| --- | --- |
| oe-NC | UUGUACUACACAAAAGUACUG |
| oe-Pro | UCCCGGACGAGCCCCCA |
| sh-NC | CAGUACUUUUGUGUAGUACAA |
| sh-Pro | UGGGGGCUCGUCCGGGA |
